# Crystal structure of 10-[(3-oxo-3*H*-benzo[*f*]chromen-1-yl)meth­yl]-2-tri­fluoro­methyl-9a,10-di­hydro­benz[4,5]imidazo[1,2-*a*]pyrimidin-4(5a*H*)-one

**DOI:** 10.1107/S2056989015014425

**Published:** 2015-08-22

**Authors:** Chandra, Shamantha Kumar., K. B. Puttaraju, K. Shivashankar, M. Mahendra

**Affiliations:** a Department of Studies in Physics, Manasagangotri, University of Mysore, Mysore 570 006, India; b Department of Physics, SJB Institute of Technology, Kengeri, Bangalore 560 060, India; c Department of Chemistry, Central College Campus, Bangalore University, Bangalore 560 001, India

**Keywords:** crystal structure, fused-ring system, chromene, benzimidazole, pyrimidinone, benzo­pyrimidine, π–π stacking inter­actions

## Abstract

In the title compound, C_25_H_14_F_3_N_3_O_3_, the dihedral angle between the planes of the benz[4,5]imidazo[1,2-*a*]pyrimidine unit (r.m.s. deviation = 0.035 Å) and the benzochromene ring system (r.m.s. deviation = 0.106 Å) is 72.82 (5)°. In the crystal, mol­ecules are linked by C—H⋯O inter­actions, generating [010] *C*(9) chains. A weak aromatic π–π stacking inter­action [centroid–centroid separation = 3.5376 (15) Å] is also observed.

## Related literature   

For background to benzo­pyrimidine derivatives, see: Bodke *et al.* (2003 ▸); Moneam *et al.* (2004 ▸). For the synthesis of the title compound, see: Puttaraju *et al.* (2013 ▸). For a related structure, see: Chandra *et al.* (2013 ▸).
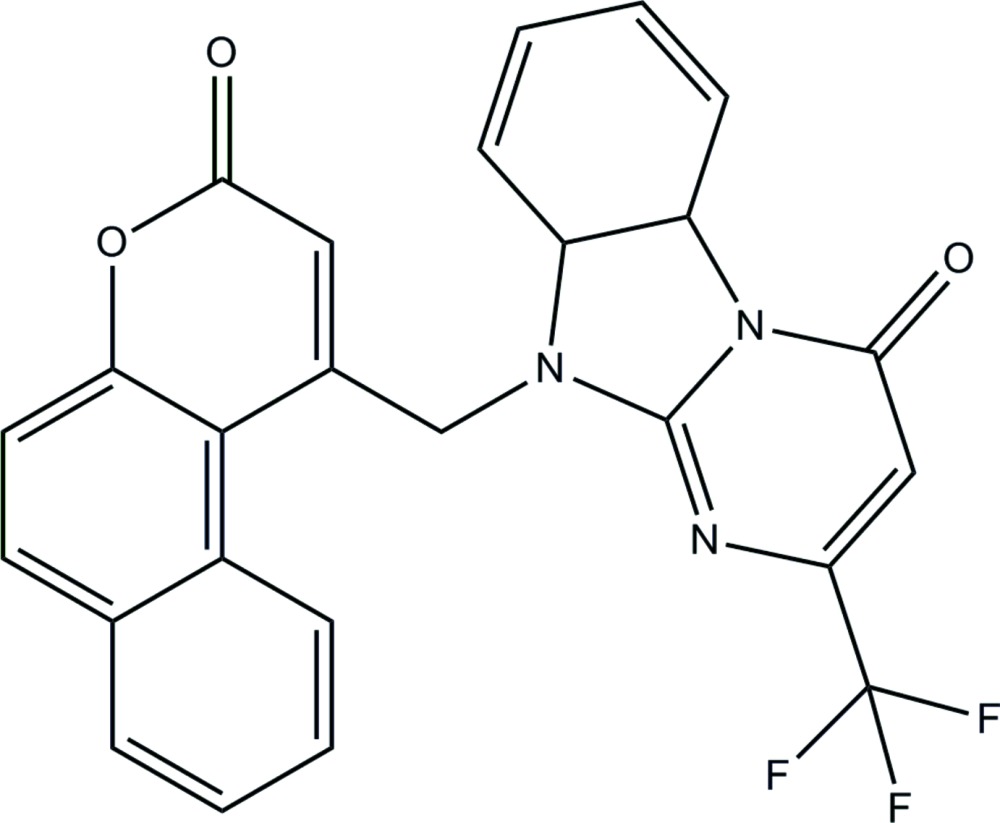


## Experimental   

### Crystal data  

C_25_H_14_F_3_N_3_O_3_
*M* *_r_* = 461.39
Monoclinic, 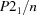

*a* = 9.7665 (5) Å
*b* = 7.7950 (4) Å
*c* = 27.0602 (16) Å
β = 95.186 (5)°
*V* = 2051.66 (19) Å^3^
*Z* = 4
Mo *K*α radiation
μ = 0.12 mm^−1^
*T* = 293 K
0.30 × 0.25 × 0.20 mm

### Data collection  

Bruker APEXII CCD area-detector diffractometer
9209 measured reflections
5020 independent reflections
2637 reflections with *I* > 2σ(*I*)
*R*_int_ = 0.033

### Refinement  

*R*[*F* ^2^ > 2σ(*F* ^2^)] = 0.057
*wR*(*F* ^2^) = 0.176
*S* = 1.00
5020 reflections
308 parameters
H-atom parameters constrained
Δρ_max_ = 0.21 e Å^−3^
Δρ_min_ = −0.19 e Å^−3^

### 

Data collection: *APEX2* (Bruker, 2009 ▸); cell refinement: *SAINT* (Bruker, 2009 ▸); data reduction: *SAINT* ; program(s) used to solve structure: *SHELXS97* (Sheldrick, 2008 ▸); program(s) used to refine structure: *SHELXL97* (Sheldrick, 2008 ▸); molecular graphics: *PLATON* (Spek, 2009 ▸); software used to prepare material for publication: *SHELXL97*.

## Supplementary Material

Crystal structure: contains datablock(s) global, I. DOI: 10.1107/S2056989015014425/hb7467sup1.cif

Structure factors: contains datablock(s) I. DOI: 10.1107/S2056989015014425/hb7467Isup2.hkl

Supporting information file. DOI: 10.1107/S2056989015014425/hb7467Isup3.cml

**Fig. 1.**
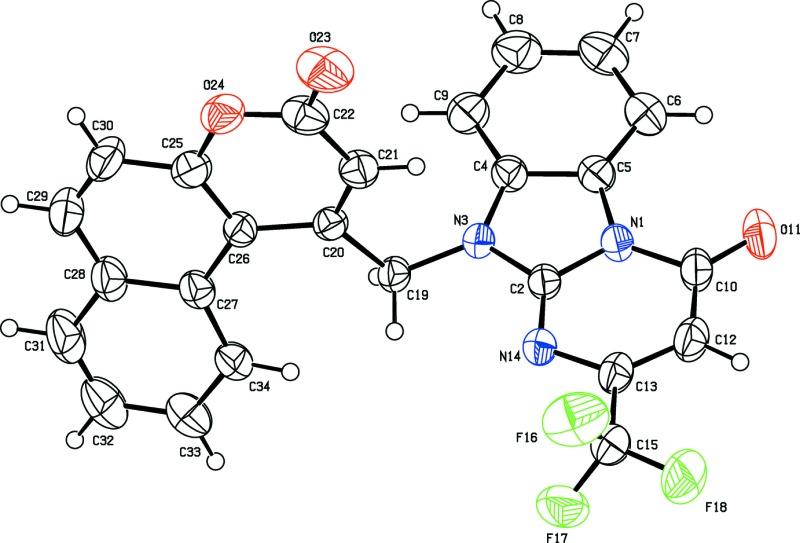
Perspective diagram of the molecule with 50% probability displacement ellipsoids.

**Fig. 2.**
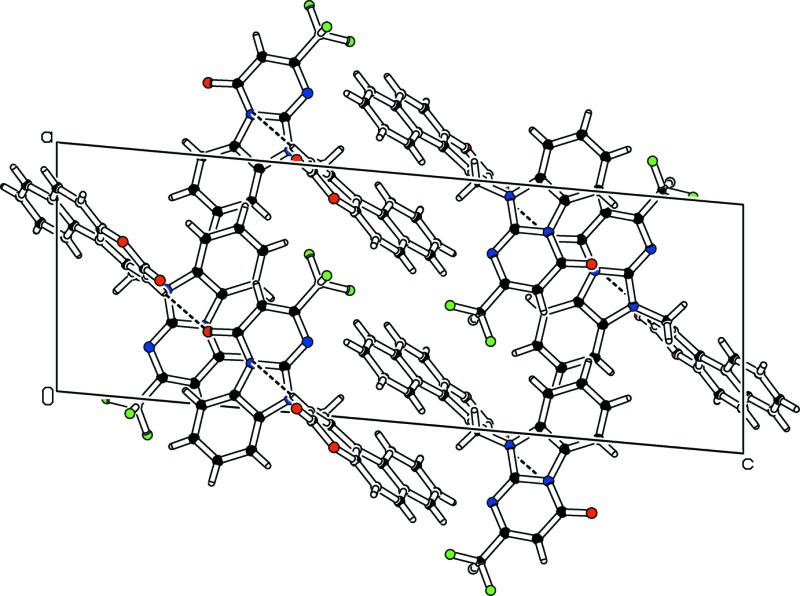
Packing diagram of the molecule viewed down the b axis.

. DOI: 10.1107/S2056989015014425/hb7467fig1.tif
Perspective diagram of the mol­ecule with 50% probability displacement ellipsoids.

b . DOI: 10.1107/S2056989015014425/hb7467fig2.tif
Packing diagram of the mol­ecule viewed down the *b* axis.

CCDC reference: 1416062

Additional supporting information:  crystallographic information; 3D view; checkCIF report

## Figures and Tables

**Table 1:** Hydrogen-bond geometry (, )

*D*H*A*	*D*H	H*A*	*D* *A*	*D*H*A*
C21H21O11^i^	0.93	2.33	3.241(3)	168

Symmetry code: (i) 
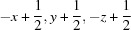
.

## supplementary crystallographic information

### S1. Comment

The heterocycles containing benzopyrimidine moiety has a variety of biological activities such as analgesic, anti-inflammatory and antimicrobial activities (Moneam *et al.*, 2004 and Bodke *et al.*, 2003). As part of our studies of these systems (Puttaraju *et al.*, 2013), the title compound was prepared and characterized by single-crystal X-ray diffraction.

In the molecular structure of the title compound (Fig. 1), the three fused rings of the benz[4,5]imidazo[1,2-*a*]pyrimidine unit are essentially coplanar; the maximum deviation from the mean plane being -0.084 (2) Å for atom O11. The dihedral angle between the three fused rings of the benz[4,5]imidazo[1,2-*a*]pyrimidine with the benzochromene moiety is 72.82 (5)°. Benzochromene moiety and fused rings of the benz[4,5]imidazo[1,2-*a*]pyrimidine derivatives bridged by the carbon atom (C19) and this inter atomic bond conformation is characterized by torsion angles of 116.5 (2)° (C2–N3–C19–C20) and 160.66 (18)° (N3–C19–C20–C26), respectively. The bond lengths and angles are generally within normal ranges and are comparable to a related structure (Chandra *et al.*, 2013). The packing diagram of the molecule exhibits chain when viewed down the *b* axis as shown in Fig. 2.

### S2. Experimental

The compound was synthesized by microwave irradiation method (Puttaraju *et al.*, 2013). The synthesized compound (yield = 93%, m.p = 228–230 °C) was recrystallized from 1:3 ethyl acetate and chloroform solution to get yellow block shaped crystals.

### S3. Refinement

H atoms were placed at idealized positions and allowed to ride on their parent atoms with C–H distances in the range of 0.93 to 0.97 Å; *U*_iso_(H) = 1.2–1.5*U*_eq_(carrier atom) for all H atoms.

### Crystal data

**Table:**

C_25_H_14_F_3_N_3_O_3_	*F*(000) = 944
*M**_r_* = 461.39	*D*_x_ = 1.494 Mg m^−^^3^
Monoclinic, *P*2_1_/*n*	Mo *K*α radiation, λ = 0.71073 Å
Hall symbol: -P 2yn	Cell parameters from 5020 reflections
*a* = 9.7665 (5) Å	θ = 2.7–28.3°
*b* = 7.7950 (4) Å	µ = 0.12 mm^−^^1^
*c* = 27.0602 (16) Å	*T* = 293 K
β = 95.186 (5)°	Bolck, yellow
*V* = 2051.66 (19) Å^3^	0.30 × 0.25 × 0.20 mm
*Z* = 4	

### Data collection

**Table:**

Bruker APEXII CCD area-detector diffractometer	*R*_int_ = 0.033
ω and φ scans	θ_max_ = 28.3°, θ_min_ = 2.7°
9209 measured reflections	*h* = −11→12
5020 independent reflections	*k* = −5→10
2637 reflections with *I* > 2σ(*I*)	*l* = −27→36

### Refinement

**Table:**

Refinement on *F*^2^	Secondary atom site location: difference Fourier map
Least-squares matrix: full	Hydrogen site location: inferred from neighbouring sites
*R*[*F*^2^ > 2σ(*F*^2^)] = 0.057	H-atom parameters constrained
*wR*(*F*^2^) = 0.176	*w* = 1/[σ^2^(*F*_o_^2^) + (0.0676*P*)^2^] where *P* = (*F*_o_^2^ + 2*F*_c_^2^)/3
*S* = 1.00	(Δ/σ)_max_ < 0.001
5020 reflections	Δρ_max_ = 0.21 e Å^−^^3^
308 parameters	Δρ_min_ = −0.19 e Å^−^^3^
0 restraints	Extinction correction: *SHELXL*, FC^*^=KFC[1+0.001XFC^2^Λ^3^/SIN(2Θ)]^-1/4^
Primary atom site location: structure-invariant direct methods	Extinction coefficient: 0.0030 (8)

### Special details

**Table:**

Geometry. Bond distances, angles *etc*. have been calculated using the rounded fractional coordinates. All su's are estimated from the variances of the (full) variance-covariance matrix. The cell e.s.d.'s are taken into account in the estimation of distances, angles and torsion angles
Refinement. Refinement on *F*^2^ for ALL reflections except those flagged by the user for potential systematic errors. Weighted *R*-factors *wR* and all goodnesses of fit *S* are based on *F*^2^, conventional *R*-factors *R* are based on *F*, with *F* set to zero for negative *F*^2^. The observed criterion of *F*^2^ > σ(*F*^2^) is used only for calculating -*R*-factor-obs *etc*. and is not relevant to the choice of reflections for refinement. *R*-factors based on *F*^2^ are statistically about twice as large as those based on *F*, and *R*-factors based on ALL data will be even larger.

### Fractional atomic coordinates and isotropic or equivalent isotropic displacement parameters (Å^2^)

**Table:**

	*x*	*y*	*z*	*U*_iso_*/*U*_eq_	
F16	−0.06281 (18)	0.8341 (3)	0.10659 (8)	0.1073 (9)	
F17	−0.01199 (16)	0.6009 (3)	0.07308 (6)	0.0921 (8)	
F18	−0.14200 (15)	0.5991 (3)	0.13215 (7)	0.1024 (9)	
O11	0.20544 (19)	0.5442 (3)	0.27911 (6)	0.0737 (8)	
O23	0.4824 (2)	1.3933 (2)	0.15054 (8)	0.0862 (9)	
O24	0.62445 (19)	1.3111 (2)	0.09616 (7)	0.0628 (7)	
N1	0.31775 (19)	0.6564 (2)	0.21634 (7)	0.0455 (6)	
N3	0.44740 (17)	0.7624 (2)	0.16034 (6)	0.0409 (6)	
N14	0.21121 (18)	0.7121 (2)	0.13481 (7)	0.0463 (6)	
C2	0.3181 (2)	0.7104 (3)	0.16781 (8)	0.0410 (7)	
C4	0.5313 (2)	0.7441 (3)	0.20479 (8)	0.0447 (7)	
C5	0.4513 (2)	0.6770 (3)	0.24038 (9)	0.0478 (8)	
C6	0.5044 (3)	0.6466 (3)	0.28881 (9)	0.0618 (9)	
C7	0.6405 (3)	0.6880 (4)	0.29999 (10)	0.0739 (11)	
C8	0.7211 (3)	0.7556 (4)	0.26516 (11)	0.0725 (11)	
C9	0.6678 (2)	0.7831 (3)	0.21638 (10)	0.0592 (9)	
C10	0.1990 (3)	0.5950 (3)	0.23595 (9)	0.0545 (8)	
C12	0.0819 (3)	0.6013 (3)	0.20043 (9)	0.0587 (9)	
C13	0.0939 (2)	0.6575 (3)	0.15344 (9)	0.0515 (8)	
C15	−0.0306 (3)	0.6693 (4)	0.11661 (10)	0.0623 (10)	
C19	0.4956 (2)	0.7898 (3)	0.11163 (8)	0.0424 (7)	
C20	0.5353 (2)	0.9744 (3)	0.10211 (8)	0.0402 (7)	
C21	0.4909 (2)	1.0969 (3)	0.13174 (9)	0.0511 (8)	
C22	0.5260 (3)	1.2748 (3)	0.12793 (10)	0.0608 (10)	
C25	0.6706 (3)	1.1898 (3)	0.06486 (9)	0.0538 (8)	
C26	0.6241 (2)	1.0222 (3)	0.06349 (8)	0.0418 (7)	
C27	0.6713 (2)	0.9122 (3)	0.02498 (8)	0.0461 (7)	
C28	0.7704 (2)	0.9786 (4)	−0.00588 (9)	0.0598 (9)	
C29	0.8182 (3)	1.1485 (4)	0.00084 (11)	0.0763 (11)	
C30	0.7689 (3)	1.2528 (4)	0.03478 (11)	0.0730 (11)	
C31	0.8178 (3)	0.8749 (5)	−0.04365 (11)	0.0803 (13)	
C32	0.7698 (3)	0.7147 (5)	−0.05239 (11)	0.0787 (13)	
C33	0.6709 (3)	0.6507 (4)	−0.02363 (10)	0.0682 (10)	
C34	0.6220 (3)	0.7459 (3)	0.01357 (9)	0.0536 (8)	
H6	0.45110	0.60090	0.31240	0.0740*	
H7	0.67990	0.66990	0.33210	0.0890*	
H8	0.81240	0.78320	0.27460	0.0870*	
H9	0.72200	0.82580	0.19270	0.0710*	
H12	−0.00350	0.56670	0.20950	0.0700*	
H19A	0.42380	0.75540	0.08650	0.0510*	
H19B	0.57470	0.71680	0.10830	0.0510*	
H21	0.43440	1.06460	0.15600	0.0610*	
H29	0.88510	1.18930	−0.01850	0.0920*	
H30	0.79970	1.36540	0.03820	0.0880*	
H31	0.88380	0.91840	−0.06300	0.0960*	
H32	0.80250	0.64810	−0.07730	0.0950*	
H33	0.63690	0.54050	−0.02970	0.0820*	
H34	0.55460	0.69940	0.03170	0.0640*	

### Atomic displacement parameters (Å^2^)

**Table:**

	*U*^11^	*U*^22^	*U*^33^	*U*^12^	*U*^13^	*U*^23^
F16	0.0900 (14)	0.0943 (15)	0.1297 (18)	0.0138 (11)	−0.0336 (12)	0.0102 (13)
F17	0.0675 (10)	0.1379 (17)	0.0697 (11)	−0.0180 (11)	−0.0005 (8)	−0.0251 (11)
F18	0.0503 (10)	0.1613 (19)	0.0968 (14)	−0.0305 (11)	0.0128 (9)	0.0081 (13)
O11	0.0810 (13)	0.0911 (15)	0.0517 (11)	−0.0130 (11)	0.0211 (9)	0.0169 (11)
O23	0.1251 (18)	0.0480 (11)	0.0847 (15)	0.0111 (11)	0.0045 (13)	−0.0181 (11)
O24	0.0774 (13)	0.0432 (9)	0.0664 (12)	−0.0115 (9)	−0.0012 (10)	0.0027 (9)
N1	0.0509 (11)	0.0471 (11)	0.0396 (10)	−0.0036 (9)	0.0106 (8)	0.0015 (9)
N3	0.0404 (10)	0.0434 (10)	0.0397 (10)	−0.0047 (9)	0.0083 (7)	−0.0002 (9)
N14	0.0429 (10)	0.0540 (12)	0.0426 (10)	−0.0056 (10)	0.0078 (8)	0.0004 (10)
C2	0.0463 (13)	0.0375 (12)	0.0402 (12)	−0.0020 (10)	0.0101 (9)	−0.0008 (10)
C4	0.0462 (13)	0.0448 (13)	0.0435 (12)	0.0003 (11)	0.0068 (10)	−0.0018 (11)
C5	0.0510 (14)	0.0490 (14)	0.0434 (12)	0.0005 (12)	0.0048 (10)	−0.0012 (12)
C6	0.0688 (17)	0.0678 (18)	0.0484 (14)	0.0022 (14)	0.0038 (12)	0.0029 (14)
C7	0.076 (2)	0.090 (2)	0.0520 (16)	0.0069 (17)	−0.0137 (14)	0.0015 (16)
C8	0.0582 (17)	0.086 (2)	0.0704 (19)	0.0014 (16)	−0.0095 (14)	−0.0032 (17)
C9	0.0480 (14)	0.0681 (17)	0.0612 (16)	−0.0014 (13)	0.0038 (12)	−0.0014 (15)
C10	0.0614 (15)	0.0549 (15)	0.0502 (14)	−0.0069 (13)	0.0210 (12)	0.0048 (13)
C12	0.0488 (14)	0.0646 (17)	0.0651 (17)	−0.0092 (13)	0.0189 (12)	0.0038 (14)
C13	0.0443 (13)	0.0544 (15)	0.0574 (15)	−0.0057 (12)	0.0136 (11)	0.0026 (13)
C15	0.0518 (16)	0.076 (2)	0.0597 (17)	−0.0108 (15)	0.0080 (12)	−0.0019 (16)
C19	0.0450 (12)	0.0405 (12)	0.0434 (12)	−0.0041 (10)	0.0128 (9)	−0.0030 (10)
C20	0.0414 (12)	0.0378 (11)	0.0409 (11)	−0.0025 (10)	0.0016 (9)	0.0000 (10)
C21	0.0587 (15)	0.0443 (13)	0.0506 (14)	0.0006 (12)	0.0070 (11)	−0.0034 (12)
C22	0.0789 (19)	0.0460 (15)	0.0558 (16)	0.0076 (14)	−0.0039 (14)	−0.0056 (13)
C25	0.0565 (15)	0.0501 (14)	0.0531 (14)	−0.0076 (12)	−0.0043 (11)	0.0045 (13)
C26	0.0403 (11)	0.0460 (13)	0.0384 (11)	−0.0023 (10)	0.0006 (9)	0.0052 (10)
C27	0.0388 (11)	0.0601 (15)	0.0391 (11)	0.0036 (11)	0.0018 (9)	0.0043 (12)
C28	0.0456 (14)	0.085 (2)	0.0498 (14)	−0.0011 (14)	0.0095 (11)	0.0087 (15)
C29	0.0701 (19)	0.097 (2)	0.0637 (19)	−0.0268 (18)	0.0162 (15)	0.0151 (18)
C30	0.0746 (19)	0.0704 (19)	0.0729 (19)	−0.0324 (16)	0.0013 (15)	0.0196 (17)
C31	0.0624 (18)	0.122 (3)	0.0605 (18)	0.006 (2)	0.0279 (14)	0.005 (2)
C32	0.077 (2)	0.108 (3)	0.0533 (17)	0.024 (2)	0.0178 (15)	−0.0043 (19)
C33	0.081 (2)	0.0726 (19)	0.0519 (15)	0.0111 (15)	0.0106 (14)	−0.0084 (15)
C34	0.0634 (15)	0.0552 (15)	0.0435 (13)	−0.0026 (13)	0.0115 (11)	−0.0066 (12)

### Geometric parameters (Å, º)

**Table:**

F16—C15	1.344 (4)	C21—C22	1.434 (3)
F17—C15	1.320 (3)	C25—C26	1.383 (3)
F18—C15	1.320 (3)	C25—C30	1.402 (4)
O11—C10	1.229 (3)	C26—C27	1.456 (3)
O23—C22	1.207 (3)	C27—C28	1.431 (3)
O24—C22	1.376 (3)	C27—C34	1.407 (3)
O24—C25	1.372 (3)	C28—C29	1.411 (4)
N1—C2	1.379 (3)	C28—C31	1.414 (4)
N1—C5	1.413 (3)	C29—C30	1.348 (4)
N1—C10	1.403 (3)	C31—C32	1.347 (5)
N3—C2	1.359 (3)	C32—C33	1.387 (4)
N3—C4	1.400 (3)	C33—C34	1.371 (4)
N3—C19	1.455 (3)	C6—H6	0.9300
N14—C2	1.311 (3)	C7—H7	0.9300
N14—C13	1.361 (3)	C8—H8	0.9300
C4—C5	1.396 (3)	C9—H9	0.9300
C4—C9	1.376 (3)	C12—H12	0.9300
C5—C6	1.386 (3)	C19—H19A	0.9700
C6—C7	1.375 (4)	C19—H19B	0.9700
C7—C8	1.386 (4)	C21—H21	0.9300
C8—C9	1.391 (4)	C29—H29	0.9300
C10—C12	1.427 (4)	C30—H30	0.9300
C12—C13	1.360 (3)	C31—H31	0.9300
C13—C15	1.504 (4)	C32—H32	0.9300
C19—C20	1.518 (3)	C33—H33	0.9300
C20—C21	1.344 (3)	C34—H34	0.9300
C20—C26	1.465 (3)		
			
F16···N14	2.878 (3)	C32···C22^iii^	3.386 (4)
F16···C27^i^	3.310 (3)	C32···C21^iii^	3.502 (4)
F17···N14	2.763 (2)	C33···C22^iii^	3.320 (4)
F17···H30^ii^	2.7100	C33···O24^iii^	3.352 (3)
F17···H29^iii^	2.5900	C33···C30^viii^	3.572 (4)
F17···H32^iv^	2.8100	C34···C19	3.045 (3)
F18···H9^i^	2.8200	C34···C25^iii^	3.439 (4)
F18···H12	2.4000	C34···C26^iii^	3.534 (3)
O11···C6	3.015 (3)	C2···H21	3.0100
O11···C2^v^	2.991 (3)	C4···H21	2.9400
O11···N1^v^	3.035 (3)	C9···H19B	3.0300
O11···N3^v^	3.191 (3)	C10···H6	3.0700
O11···C21^v^	3.241 (3)	C19···H9	2.9800
O11···C4^v^	3.345 (3)	C19···H34	2.3900
O11···C5^v^	3.266 (3)	C20···H34	2.8900
O23···N3^vi^	2.912 (2)	C22···H7^vii^	3.0900
O23···C2^vi^	3.006 (3)	C22···H8^vii^	2.9500
O23···N1^vi^	3.237 (3)	C25···H7^vii^	3.0300
O23···C5^vi^	3.321 (3)	C27···H19B	2.9500
O23···C19^vi^	3.271 (3)	C30···H33^vi^	3.0500
O23···C4^vi^	3.120 (3)	C33···H30^viii^	2.9900
O24···C33^iii^	3.352 (3)	C34···H19B	2.6600
O11···H6	2.5200	C34···H19A	2.8900
O11···H21^v^	2.3300	H6···O11	2.5200
O23···H8^vii^	2.8500	H6···C10	3.0700
O24···H7^vii^	2.8200	H7···O24^xi^	2.8200
N1···O23^viii^	3.237 (3)	H7···C22^xi^	3.0900
N1···O11^ix^	3.035 (3)	H7···C25^xi^	3.0300
N3···O23^viii^	2.912 (2)	H8···O23^xi^	2.8500
N3···O11^ix^	3.191 (3)	H8···C22^xi^	2.9500
N14···F17	2.763 (2)	H9···F18^x^	2.8200
N14···F16	2.878 (3)	H9···C19	2.9800
N3···H21	2.3600	H12···F18	2.4000
N14···H19A	2.5700	H19A···N14	2.5700
C2···O23^viii^	3.006 (3)	H19A···C34	2.8900
C2···O11^ix^	2.991 (3)	H19A···H34	2.0900
C4···C21	3.389 (3)	H19B···C9	3.0300
C4···O23^viii^	3.120 (3)	H19B···C27	2.9500
C4···O11^ix^	3.345 (3)	H19B···C34	2.6600
C5···O11^ix^	3.266 (3)	H19B···H34	2.0700
C5···O23^viii^	3.321 (3)	H21···N3	2.3600
C6···O11	3.015 (3)	H21···C2	3.0100
C9···C20	3.569 (3)	H21···C4	2.9400
C19···C34	3.045 (3)	H21···O11^ix^	2.3300
C19···O23^viii^	3.271 (3)	H29···H31	2.4300
C20···C9	3.569 (3)	H29···F17^iii^	2.5900
C21···O11^ix^	3.241 (3)	H30···F17^xii^	2.7100
C21···C4	3.389 (3)	H30···C33^vi^	2.9900
C21···C32^iii^	3.502 (4)	H31···H29	2.4300
C22···C33^iii^	3.320 (4)	H32···F17^iv^	2.8100
C22···C32^iii^	3.386 (4)	H33···C30^viii^	3.0500
C25···C34^iii^	3.439 (4)	H34···C19	2.3900
C26···C34^iii^	3.534 (3)	H34···C20	2.8900
C27···F16^x^	3.310 (3)	H34···H19A	2.0900
C30···C33^vi^	3.572 (4)	H34···H19B	2.0700
			
C22—O24—C25	122.07 (19)	C20—C26—C25	115.8 (2)
C2—N1—C5	108.78 (18)	C20—C26—C27	127.4 (2)
C2—N1—C10	122.53 (19)	C25—C26—C27	116.8 (2)
C5—N1—C10	128.7 (2)	C26—C27—C28	118.6 (2)
C2—N3—C4	108.75 (17)	C26—C27—C34	125.1 (2)
C2—N3—C19	124.04 (17)	C28—C27—C34	116.2 (2)
C4—N3—C19	125.53 (16)	C27—C28—C29	119.9 (2)
C2—N14—C13	112.93 (19)	C27—C28—C31	119.9 (3)
N1—C2—N3	108.36 (17)	C29—C28—C31	120.3 (2)
N1—C2—N14	125.54 (19)	C28—C29—C30	121.3 (3)
N3—C2—N14	126.1 (2)	C25—C30—C29	119.3 (3)
N3—C4—C5	108.08 (17)	C28—C31—C32	121.7 (3)
N3—C4—C9	130.8 (2)	C31—C32—C33	118.9 (3)
C5—C4—C9	121.1 (2)	C32—C33—C34	121.6 (3)
N1—C5—C4	106.02 (19)	C27—C34—C33	121.7 (2)
N1—C5—C6	131.8 (2)	C5—C6—H6	122.00
C4—C5—C6	122.1 (2)	C7—C6—H6	122.00
C5—C6—C7	116.1 (2)	C6—C7—H7	119.00
C6—C7—C8	122.5 (3)	C8—C7—H7	119.00
C7—C8—C9	121.1 (3)	C7—C8—H8	119.00
C4—C9—C8	117.0 (2)	C9—C8—H8	119.00
O11—C10—N1	119.8 (2)	C4—C9—H9	122.00
O11—C10—C12	128.3 (3)	C8—C9—H9	121.00
N1—C10—C12	111.9 (2)	C10—C12—H12	120.00
C10—C12—C13	120.6 (2)	C13—C12—H12	120.00
N14—C13—C12	126.4 (2)	N3—C19—H19A	109.00
N14—C13—C15	113.1 (2)	N3—C19—H19B	109.00
C12—C13—C15	120.4 (2)	C20—C19—H19A	109.00
F16—C15—F17	104.7 (2)	C20—C19—H19B	109.00
F16—C15—F18	106.0 (2)	H19A—C19—H19B	108.00
F16—C15—C13	110.7 (2)	C20—C21—H21	118.00
F17—C15—F18	107.5 (2)	C22—C21—H21	118.00
F17—C15—C13	113.5 (2)	C28—C29—H29	119.00
F18—C15—C13	113.8 (2)	C30—C29—H29	119.00
N3—C19—C20	113.66 (18)	C25—C30—H30	120.00
C19—C20—C21	118.11 (19)	C29—C30—H30	120.00
C19—C20—C26	122.60 (19)	C28—C31—H31	119.00
C21—C20—C26	119.3 (2)	C32—C31—H31	119.00
C20—C21—C22	123.5 (2)	C31—C32—H32	121.00
O23—C22—O24	117.3 (2)	C33—C32—H32	121.00
O23—C22—C21	127.3 (3)	C32—C33—H33	119.00
O24—C22—C21	115.4 (2)	C34—C33—H33	119.00
O24—C25—C26	122.8 (2)	C27—C34—H34	119.00
O24—C25—C30	113.4 (2)	C33—C34—H34	119.00
C26—C25—C30	123.8 (2)		
			
C22—O24—C25—C30	178.8 (2)	C10—C12—C13—C15	−178.0 (2)
C25—O24—C22—O23	−173.1 (2)	C10—C12—C13—N14	0.0 (4)
C25—O24—C22—C21	9.3 (3)	N14—C13—C15—F16	−68.0 (3)
C22—O24—C25—C26	−1.3 (4)	C12—C13—C15—F18	−9.0 (4)
C5—N1—C2—N14	−178.9 (2)	N14—C13—C15—F17	49.4 (3)
C10—N1—C2—N3	179.82 (18)	N14—C13—C15—F18	172.8 (2)
C10—N1—C2—N14	0.3 (3)	C12—C13—C15—F16	110.3 (3)
C5—N1—C2—N3	0.7 (2)	C12—C13—C15—F17	−132.3 (3)
C2—N1—C10—O11	178.1 (2)	N3—C19—C20—C26	160.66 (18)
C2—N1—C10—C12	−1.9 (3)	N3—C19—C20—C21	−16.8 (3)
C5—N1—C10—O11	−3.0 (4)	C19—C20—C21—C22	177.3 (2)
C2—N1—C5—C4	−0.2 (2)	C21—C20—C26—C27	−173.5 (2)
C2—N1—C5—C6	178.2 (2)	C19—C20—C26—C25	−169.1 (2)
C10—N1—C5—C4	−179.3 (2)	C19—C20—C26—C27	9.1 (3)
C10—N1—C5—C6	−0.9 (4)	C26—C20—C21—C22	−0.2 (3)
C5—N1—C10—C12	177.1 (2)	C21—C20—C26—C25	8.3 (3)
C19—N3—C2—N14	−15.4 (3)	C20—C21—C22—O23	174.1 (3)
C2—N3—C4—C9	−178.1 (2)	C20—C21—C22—O24	−8.6 (4)
C19—N3—C4—C5	−164.93 (19)	O24—C25—C26—C20	−7.7 (3)
C2—N3—C4—C5	0.7 (2)	O24—C25—C26—C27	173.9 (2)
C2—N3—C19—C20	116.5 (2)	C30—C25—C26—C20	172.2 (2)
C4—N3—C19—C20	−80.0 (2)	C30—C25—C26—C27	−6.2 (4)
C19—N3—C2—N1	165.06 (18)	O24—C25—C30—C29	−177.2 (3)
C19—N3—C4—C9	16.2 (4)	C26—C25—C30—C29	2.9 (4)
C4—N3—C2—N1	−0.8 (2)	C20—C26—C27—C28	−173.0 (2)
C4—N3—C2—N14	178.7 (2)	C20—C26—C27—C34	10.4 (4)
C13—N14—C2—N3	−178.0 (2)	C25—C26—C27—C28	5.2 (3)
C2—N14—C13—C12	−1.6 (3)	C25—C26—C27—C34	−171.4 (2)
C2—N14—C13—C15	176.5 (2)	C26—C27—C28—C29	−1.2 (3)
C13—N14—C2—N1	1.5 (3)	C26—C27—C28—C31	−179.8 (2)
N3—C4—C9—C8	177.6 (2)	C34—C27—C28—C29	175.7 (2)
C5—C4—C9—C8	−1.2 (4)	C34—C27—C28—C31	−2.9 (3)
N3—C4—C5—N1	−0.3 (2)	C26—C27—C34—C33	179.4 (2)
N3—C4—C5—C6	−178.9 (2)	C28—C27—C34—C33	2.7 (4)
C9—C4—C5—N1	178.7 (2)	C27—C28—C29—C30	−2.3 (4)
C9—C4—C5—C6	0.1 (4)	C31—C28—C29—C30	176.3 (3)
C4—C5—C6—C7	0.6 (4)	C27—C28—C31—C32	1.5 (4)
N1—C5—C6—C7	−177.6 (2)	C29—C28—C31—C32	−177.1 (3)
C5—C6—C7—C8	−0.3 (4)	C28—C29—C30—C25	1.6 (4)
C6—C7—C8—C9	−0.8 (5)	C28—C31—C32—C33	0.3 (5)
C7—C8—C9—C4	1.5 (4)	C31—C32—C33—C34	−0.5 (5)
O11—C10—C12—C13	−178.2 (3)	C32—C33—C34—C27	−1.1 (4)
N1—C10—C12—C13	1.7 (3)		

Symmetry codes: (i) *x*−1, *y*, *z*; (ii) *x*−1, *y*−1, *z*; (iii) −*x*+1, −*y*+2, −*z*; (iv) −*x*+1, −*y*+1, −*z*; (v) −*x*+1/2, *y*−1/2, −*z*+1/2; (vi) *x*, *y*+1, *z*; (vii) −*x*+3/2, *y*+1/2, −*z*+1/2; (viii) *x*, *y*−1, *z*; (ix) −*x*+1/2, *y*+1/2, −*z*+1/2; (x) *x*+1, *y*, *z*; (xi) −*x*+3/2, *y*−1/2, −*z*+1/2; (xii) *x*+1, *y*+1, *z*.

### Hydrogen-bond geometry (Å, º)

**Table:**

*D*—H···*A*	*D*—H	H···*A*	*D*···*A*	*D*—H···*A*
C21—H21···O11^ix^	0.93	2.33	3.241 (3)	168

Symmetry code: (ix) −*x*+1/2, *y*+1/2, −*z*+1/2.
