# Supplementary figures and images for: Crystal structure of 10-[(3-oxo-3H-benzo[f]chromen-1-yl)meth­yl]-2-tri­fluoro­methyl-9a,10-di­hydro­benz[4,5]imidazo[1,2-a]pyrimidin-4(5aH)-one
Source: Acta Crystallogr E Crystallogr Commun. 2015 Aug 22;71(Pt 9):o672–3. doi: 10.1107/S2056989015014425 (PMC4555409; doi:10.1107/S2056989015014425)

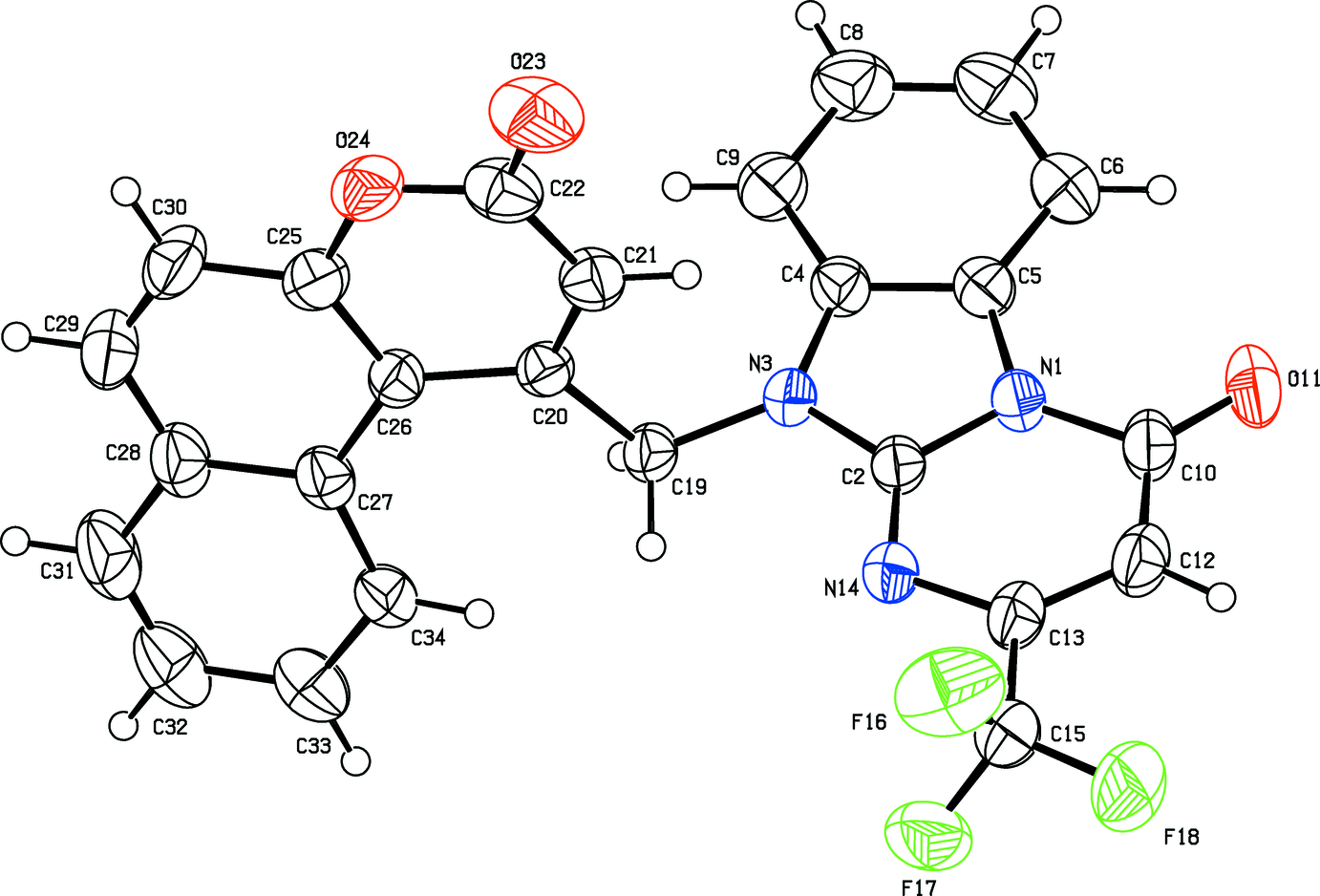

Supplement: Supplementary file 4 [file e-71-0o672-fig1.tif]

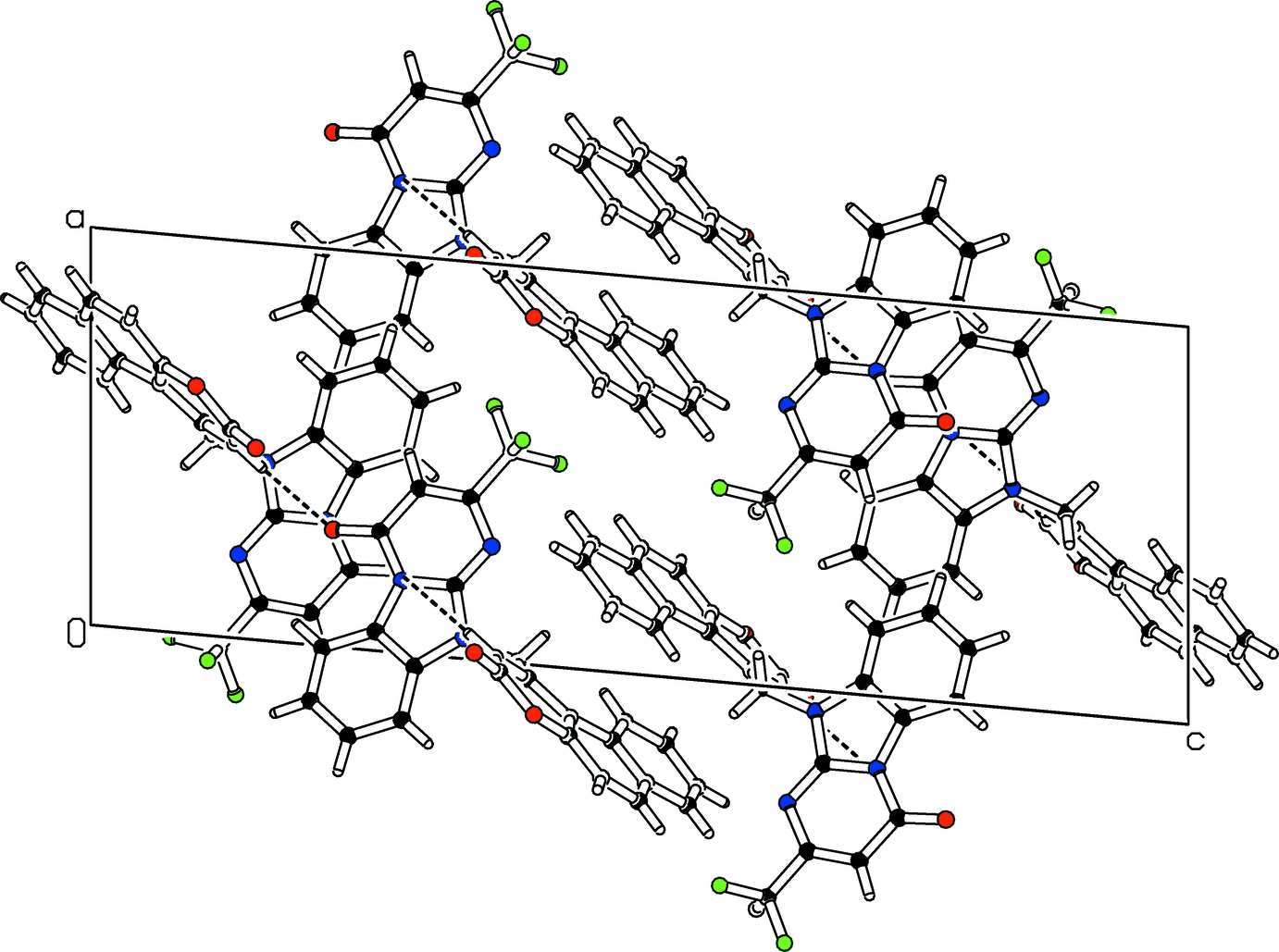

Supplement: Supplementary file 5 [file e-71-0o672-fig2.tif]
